# Bisoprolol transdermal patch for perioperative care of non-cardiac surgery in patients with hypertrophic obstructive cardiomyopathy

**DOI:** 10.1186/s12872-019-01274-6

**Published:** 2019-12-30

**Authors:** Yoichi Imori, Hitoshi Takano, Hiroshi Mase, Junya Matsuda, Hideto Sangen, Yuki Izumi, Yukichi Tokita, Takeshi Yamamoto, Wataru Shimizu

**Affiliations:** 1 Department of Cardiovascular Medicine, Nippon Medical School, 1-1-5 Sendagi, Bunkyoku, Tokyo, 113-8603 Japan; 2 Department of Anesthesiology, Department of Surgical Intensive Care, Nippon Medical School, 1-1-5 Sendagi, Bunkyoku, Tokyo, 113-8603 Japan; 3 Devision of Cardiovascular Intensive Care, Nippon Medical School, 1-1-5 Sendagi, Bunkyoku, Tokyo, 113-8603 Japan

**Keywords:** Bisoprolol transdermal patch, β-Blocker, Retrospective study, Cardiovascular complications, Hypertrophic obstructive cardiomyopathy, Hypertrophic cardiomyopathy, Non-cardiac surgery, Perioperative care

## Abstract

**Background:**

Non-cardiac surgery for hypertrophic obstructive cardiomyopathy (HOCM) is considered to require meticulous perioperative care. β-blockers are considered the first-line drugs for patients with HOCM, and they play a key role in preventing cardiovascular complications in perioperative care. The bisoprolol transdermal patch has recently become available in Japan, and it is useful for patients who are unable to take oral medication during perioperative care. The aim of this case series was to assess the hemodynamic features of patients with HOCM who used the bisoprolol transdermal patch during perioperative care for non-cardiac surgery.

**Methods:**

Between August 2016 and August 2018, we retrospectively analyzed 10 consecutive cases of HOCM with the patients using the bisoprolol transdermal patch during perioperative care. Hemodynamic and echocardiographic features were evaluated before and after patients were switched from oral bisoprolol to transdermal patch therapy or started transdermal patch therapy as a new β-blocker medication. In addition, cardiovascular complications (all-cause death, cardiac death, heart failure, ventricular tachycardia, and ventricular fibrillation) during the perioperative period were evaluated.

**Results:**

There was no significant change in the patients’ heart rate, blood pressure, ejection fraction, and pressure gradient in the left ventricle after switching from oral bisoprolol to the transdermal patch therapy. On the other hand, patients who started using the bisoprolol transdermal patch as a new ß-blocker medication tended to have a decreased heart rate and pressure gradient thereafter, but there was no significant difference in blood pressure or ejection fraction. No cardiovascular complications occurred during the perioperative period.

**Conclusions:**

We described the utilization of the bisoprolol transdermal patch during perioperative care for non-cardiac surgery in patients with HOCM. We determined that the hemodynamic features of these patients did not change significantly after switching to patch therapy. Further, initiation of the bisoprolol transdermal patch as a new ß-blocker medication sufficiently tended to decrease the pressure gradient. This unique approach can be an alternate treatment option for HOCM.

**Trial registration:**

The registry was registered in the University Hospital Medical Information Network Clinical Trials Registry (UMIN000036703). The date of registration was 10/5/2019 and it was “Retrospectively registered”.

## Background

Hypertrophic cardiomyopathy (HCM) is a complex and relatively common genetic cardiomyopathy characterized by remarkable thickness of the left ventricular (LV) wall, which is not solely explained by hypertensive heart disease or heart valve disease [1–3].

Resting- or exercise-provoked LV outflow tract (LVOT) obstruction, which primarily develop various symptoms, is present in most patients with HCM [4]. A third of the patients with HCM exhibit evidence of LVOT obstruction at rest, whereas another one-third have latent LVOT obstruction [2, 3]. Hypertrophic obstructive cardiomyopathy (HOCM) manifests with specific and dynamic hemodynamics, and careful periprocedural management is required for patients undergoing non-cardiac surgery [2, 3, 5]. Patients with outflow stenosis are considered to have more perioperative complications than those without obstruction [6].

β-blockers, which inhibit increases of LV contraction, heart rate, and the LV pressure gradient during stress, are used as first-line drugs for patients with HOCM [7]. Moreover, guidelines recommended the continuation of β-blockers in periprocedural management for the prevention of cardiovascular complications, although evidence in a large sample is still lacking [3, 5]. However, the management of patients who are unable to take β-blockers orally in the perioperative period is difficult. Recently, in addition to existing oral and intravenous β-blockers, the bisoprolol transdermal patch, which is a percutaneously absorbed β-1-blocker (Bisono® Tape; Toa Eiyo, Tokyo, Japan), has become available clinically as a new treatment option in Japan [8, 9]. Nevertheless, the transdermal drug delivery system has not been widely used in cardiovascular treatment despite its many advantages.

The aim of this case series was to evaluate the hemodynamic features of patients with HOCM who used the bisoprolol transdermal patch during perioperative care for non-cardiac surgery.

## Methods

### Study population

Ten consecutive patients with HOCM were enrolled from the Department of Cardiovascular Medicine of our institute from August 2016 and August 2018. Before non-cardiac surgery, we reviewed and optimized the medications prescribed to the patients. When non-cardiac surgery could be postponed for a few months, percutaneous transluminal septal myocardial ablation (PTSMA) or surgical septal myectomy was considered for patients if their symptoms were lifestyle-limiting after optimization of their medication and if resting or a provoked pressure gradient > 50 mmHg was confirmed by at least one method. We excluded patients who exhibited only sigmoid septum but without significant hypertrophy. We treated patients with HCM with mid-ventricular obstruction similar to those with LVOT obstruction using the bisoprolol transdermal patch.

Patients in the current study were retrospectively registered. The registry was registered in the University Hospital Medical Information Network Clinical Trials Registry (UMIN000036703). Written informed consent was obtained from all patients, and ethical approval was obtained from the review committee of Nippon Medical School (approval number 28–07-615), and the study design conformed to the ethical principles of the Declaration of Helsinki.

### Diagnosis of HCM/HOCM

We diagnosed HCM based on the presence of maximal LV wall thickness ≥ 15 mm by transthoracic echocardiography (TTE) and cardiac magnetic resonance imaging (MRI) according to the guidelines of the American College of Cardiology Foundation/American Heart Association [2]. Further, we confirmed the absence of other conditions that may explain LV hypertrophy during the clinical course of the patients. In this study, a genetic diagnosis and an endomyocardial biopsy for histological assessment were not mandatory for the diagnosis of clinical HCM. HOCM was characterized with an intraventricular pressure gradient of ≥30 mmHg on TTE at rest or on provocation. For the evaluation of the hemodynamic state of intraventricular obstruction, our laboratory staff performed stress echocardiography via supine bicycle ergometry based on a protocol described in a previous study, with some modifications [2, 10]. A symptom-limited supine bicycle protocol started at a workload of 25 watts and increased by 25-watt increments every 3 min until an endpoint was achieved. We were able to evaluate the pressure gradient continuously.

### Clinical and TTE evaluation

#### Patients who switched from oral bisoprolol to the bisoprolol transdermal patch

The patches were applied once a day to the chest, upper back, or upper arm. The antihypertensive and heart rate-lowering effects of the 8-mg bisoprolol transdermal patch are similar to those of a 5-mg oral tablet of bisoprolol fumarate, and these effects are dose-dependent in the 2–8-mg range of the bisoprolol transdermal patch (the 4-mg bisoprolol transdermal patch is considered to correspond to the 2.5-mg oral tablet) [8, 9]. We switched the medication from oral bisoprolol to the bisoprolol transdermal patch in accordance with this regimen.

#### Patients who started the bisoprolol transdermal patch as new ß-blocker medication

The patches were applied once a day to the chest, upper back, or upper arm too. Patients with HOCM received the bisoprolol transdermal patch for the first time when they were reaching their maximum dose.

We routinely conducted examinations of their symptoms, heart rate, and blood pressure, and TTE was performed before and after switching therapy from oral bisoprolol to the bisoprolol transdermal patch. In the TTE study, the resting pressure gradient by Doppler echocardiography, which is used to evaluate hemodynamic changes, was assessed as well as LV end-diastolic diameter, LV end-systolic diameter, left atrial diameter, interventricular septal thickness, posterior wall thickness, and ejection fraction (EF).

### Cardiovascular complications during the perioperative period

Cardiovascular complications during the perioperative period included all-cause death, cardiac death, heart failure, ventricular tachycardia, and ventricular fibrillation. We evaluated the anesthetic record during non-cardiac surgery, and all patients in the present study were assessed by continuous electrocardiographic monitoring during the perioperative period. We also assessed appropriate and inappropriate discharges of defibrillators by the device’s memory in patients with an implanted defibrillator. Cardiac death and heart failure were defined as per the report by the American College of Cardiology/American Heart Association task force on clinical data standards [11].

### Statistical analysis

Continuous variables are presented as medians with interquartile ranges, and categorical variables are expressed as prevalence rates (%). Continuous variables were compared using Mann-Whitney’s U-test for analysis of unpaired comparisons. Paired changes in continuous variables were analyzed using Wilcoxon’s signed-rank test. All statistical analyses were performed using SPSS Statistics 23.0 (IBM Corp., Armonk, NY, USA), and a two-sided *p*-value < 0.05 was considered statistically significant.

## Results

Ten patients with HOCM underwent non-cardiac surgery during the study period. All surgeries were performed under total anesthesia. Eight patients had LV outflow obstruction, and 2 patients had mid-ventricular obstruction. Four patients who had already undergone PTSMA were included. Their median age was 75 (interquartile range [IQR]: 58–80) years, and 7 (70%) were women. Six patients were switched from oral bisoprolol to the bisoprolol transdermal patch in preparation for non-cardiac surgery, while 4 patients started treatment with the bisoprolol transdermal patch as a new β-blocker medication. The baseline characteristics, type of surgery performed, surgical risk estimate, symptoms, and echocardiographic parameters are shown in Table 1 [12, 13]. Details of each patient are shown in the Additional file 1.

**Table 1:** Baseline characteristics

	Switching therapy group	Add-on therapy group
	*n* = 6	*n* = 4
Age (years)	72 ± 13	68 ± 14
Sex (male:female)	3 (50):3 (50)	0 (0):4 (100)
Type of surgery^a^		
Intraperitoneal	2	1
Orthopedic	2	1
Peripheral Vascular	–	1
Lung	1	–
Urological	1	–
Thyroid	–	1
Surgical risk^b^		
High	1 (17)	1 (25)
Intermediate	5 (83)	1 (25)
Low	0 (0)	2 (50)
Oral β-blocker		
Bisoprolol (7.5 mg)	1 (17)	–
Bisoprolol (5 mg)	5 (83)	–
None	–	4 (100)
Bisoprolol transdermal patch		
8 mg	6 (100)	1 (25)
4 mg	0 (0)	3 (75)
Class IA agents^c^	5 (83)	4 (100)
Class III agents	0 (0)	0 (0)
Calcium-channel blockers	5 (83)	1 (25)
Diuretics	1 (17)	0 (0)
Height (cm)	156 ± 11	155 ± 5
Weight (kg)	59 ± 15	57 ± 10
BMI (kg/m^2^)	24 ± 4	24 ± 3
NYHA class		
I	2 (33)	1 (25)
IIs	2 (33)	3 (75)
IIm	1 (17)	0 (0)
III	1 (17)	0 (0)
IV	0 (0)	0 (0)
Type of obstruction		
LVOTO	5 (83)	3 (75)
MVO	1 (17)	1 (25)
Prior PTSMA	4 (67)	0 (0)
FH-SCD	1 (17)	0 (0)
VT/Vf	0 (0)	0 (0)
Af	3 (50)	0 (1)
Prior pacemaker/ICD	1 (17)	0 (2)
Measurements of the left ventricle		
IVST (mm)	15 ± 1	15 ± 5
PWT (mm)	12 ± 2	12 ± 2
LAD (mm)	42 ± 8	37 ± 2
LVDd (mm)	44 ± 8	40 ± 2
LVDs (mm)	28 ± 9	22 ± 1

*BMI* body mass index, *NYHA* New York Heart Association, *LVOT* left ventricular outflow obstruction, *MVO* mid-ventricular obstruction, *PTSMA* percutaneous transluminal septal myocardial ablation, *FH* family history, *SCD* sudden cardiac death, *VT/Vf* ventricular tachycardia/ventricular fibrillation, *Af* atrial fibrillation, *ICD* implanted cardioverter defibrillator, *IVST* interventricular septal thickness, *PWT* posterior wall thickness, *LAD* left atrial diameter, *LVDd* left ventricular end-diastolic diameter, *LVDs* left ventricular end-systolic diameter, *EF* ejection fraction

^a^The type of surgery is presented as per the European Society of Cardiology and the European Society of Anaesthesiology guideline [13].

^b^The surgical risk estimate was a broad approximation of the 30-day risk of cardiovascular death and myocardial infarction that considers only the specific surgical intervention, without considering the patient’s comorbidities [12].

^c^Cibenzoline (100–300 mg)

### Patients who switched from oral bisoprolol to the bisoprolol transdermal patch

**Fig. 1:**
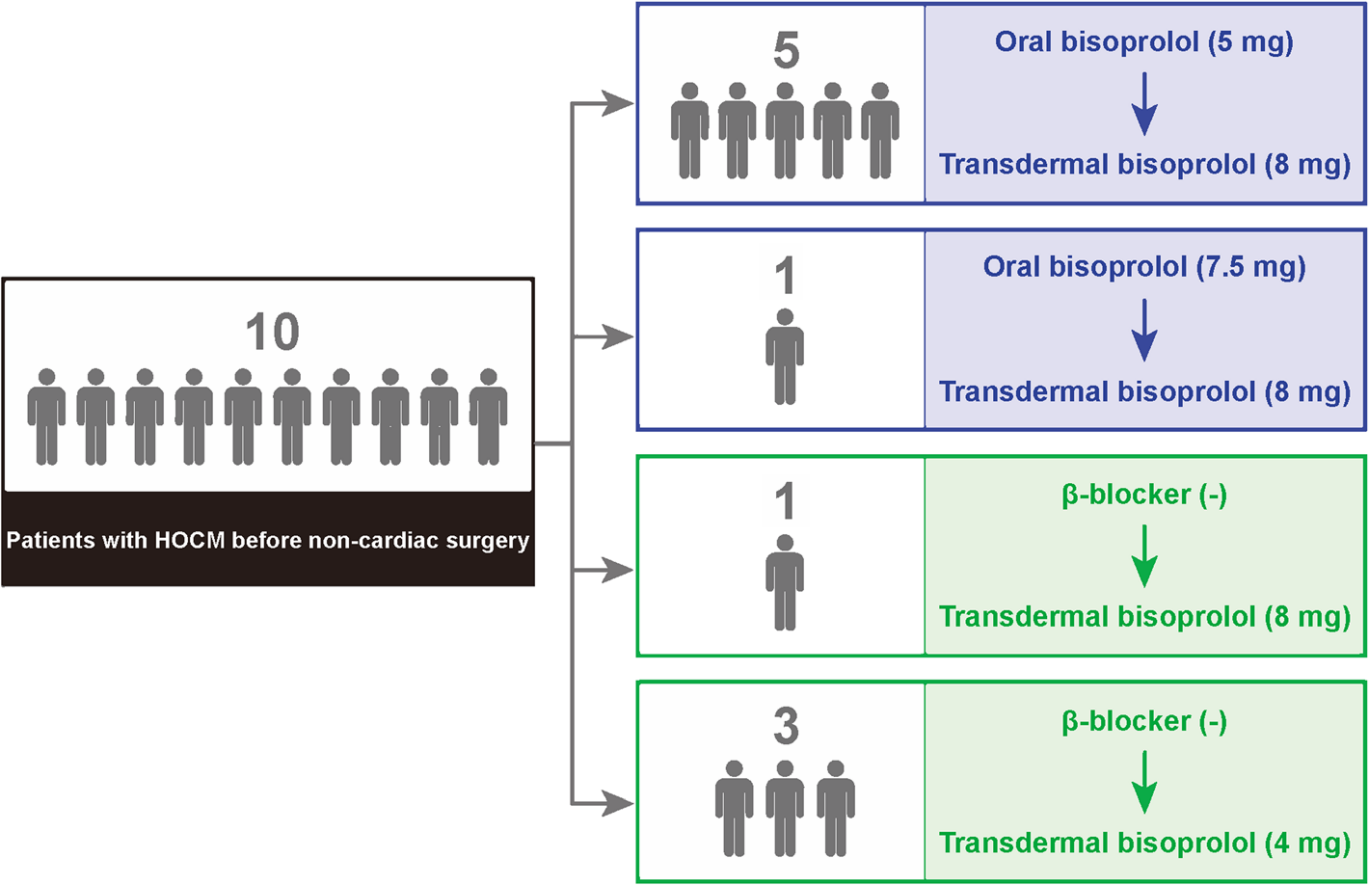
Flow chart of patients and medications. 1) Switching therapy group (blue): patients who switched from oral bisoprolol to the bisoprolol transdermal patch. We switched the medication from oral bisoprolol (5 mg) to the bisoprolol transdermal patch (8 mg) in our study. However, 1 patient had already taken oral bisoprolol (7.5 mg) and was switched to the bisoprolol transdermal patch (8 mg). 2) Add-on therapy group (green): patients who started the bisoprolol transdermal patch as a new medication. One patient was newly introduced to the 8-mg bisoprolol transdermal patch, and 3 patients were introduced to the 4-mg bisoprolol transdermal patch. HOCM = hypertrophic obstructive cardiomyopathy

**Fig. 2:**
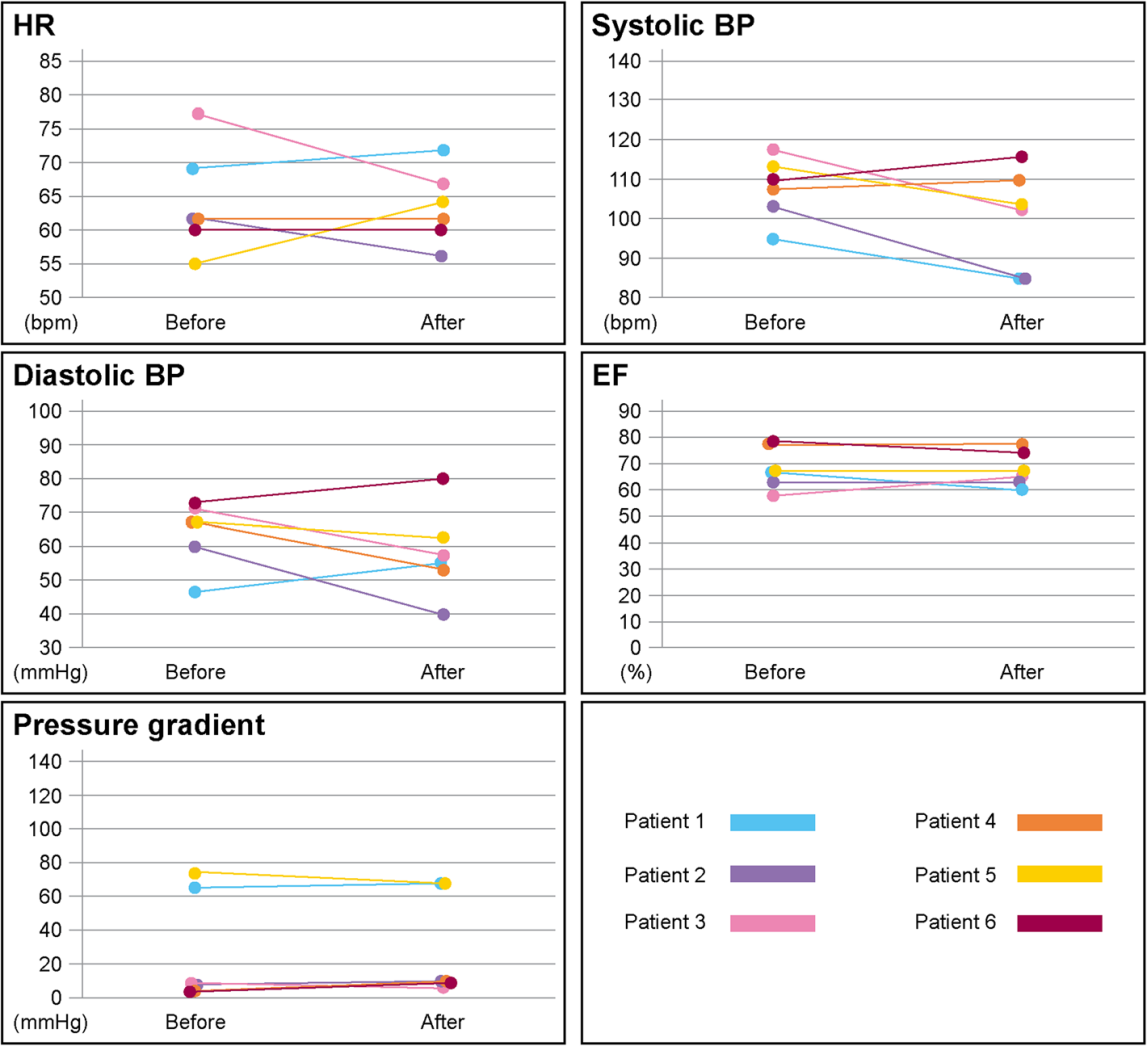
Changes of hemodynamics and echocardiographic features before and after changing the medication. In the switching therapy group, the medication was switched from 5-mg oral bisoprolol to the 8-mg bisoprolol transdermal patch in 5 cases (patient 1, patient 2, patient 3, patient 4, and patient 6). One patient who had already taken 7.5-mg oral bisoprolol (patient 5) was switched to the 8-mg bisoprolol transdermal patch. Patient 2 presented with symptomatic hypotension, so we reduced the dose of the bisoprolol transdermal patch from 8 mg to 4 mg. The systolic blood pressure of patient 1 decreased under 90 mmHg, but there was no symptom of worsened end-organ perfusion. HR = heart rate; BP = blood pressure; EF = ejection fraction; bpm = beats per minute

The medication was switched from 5-mg oral bisoprolol to the 8-mg bisoprolol transdermal patch in 5 patients (Fig. 1). In 1 patient, who had already taken 7.5-mg oral bisoprolol, the medication was switched to the 8-mg transdermal patch (Fig. 1). The changes of pulse rate, blood pressure, LV contraction, and LV pressure gradient by echocardiography before and after changing the medication are shown in the Fig. 2.

The 6 patients who switched the medication from oral bisoprolol to the bisoprolol transdermal patch demonstrated no significant difference in any parameters before and after the change in medication: heart rate, from 62 (IQR: 59–71) to 63 (IQR: 59–68) beats/min [bpm] (*p* = 0.72); systolic blood pressure/diastolic blood pressure, from 109 (IQR: 102–114)/68 (IQR: 57–71) to 103 (IQR: 85–112)/57 (IQR: 51–67) mmHg (*p* = 0.12/*p* = 0.25); left ventricular ejection fraction (LVEF), from 68 (IQR: 62–78) to 67 (IQR: 62–75)% (*p* = 0.59); and pressure gradient, from 9 (IQR: 6–68) to 9 (IQR: 9–69) mmHg (*p* = 0.52) (Fig. 2). However, in 1 case (patient 2 in Fig. 2), symptomatic hypotension occurred, so we reduced the dose of the bisoprolol transdermal patch from 8 mg to 4 mg. The heart rate of the patient who switched from 7.5-mg oral bisoprolol to the 8-mg transdermal patch was increased before and after changing the medication (patient 1 in Fig. 2).

### Patients who started the bisoprolol transdermal patch as new β-blocker medication

One patient was newly introduced to the 8-mg bisoprolol transdermal patch and 3 patients were newly introduced to the 4-mg bisoprolol transdermal patch, because they were newly diagnosed as having HOCM in the screening before non-cardiac surgery (Fig. 1).

**Fig. 3:**
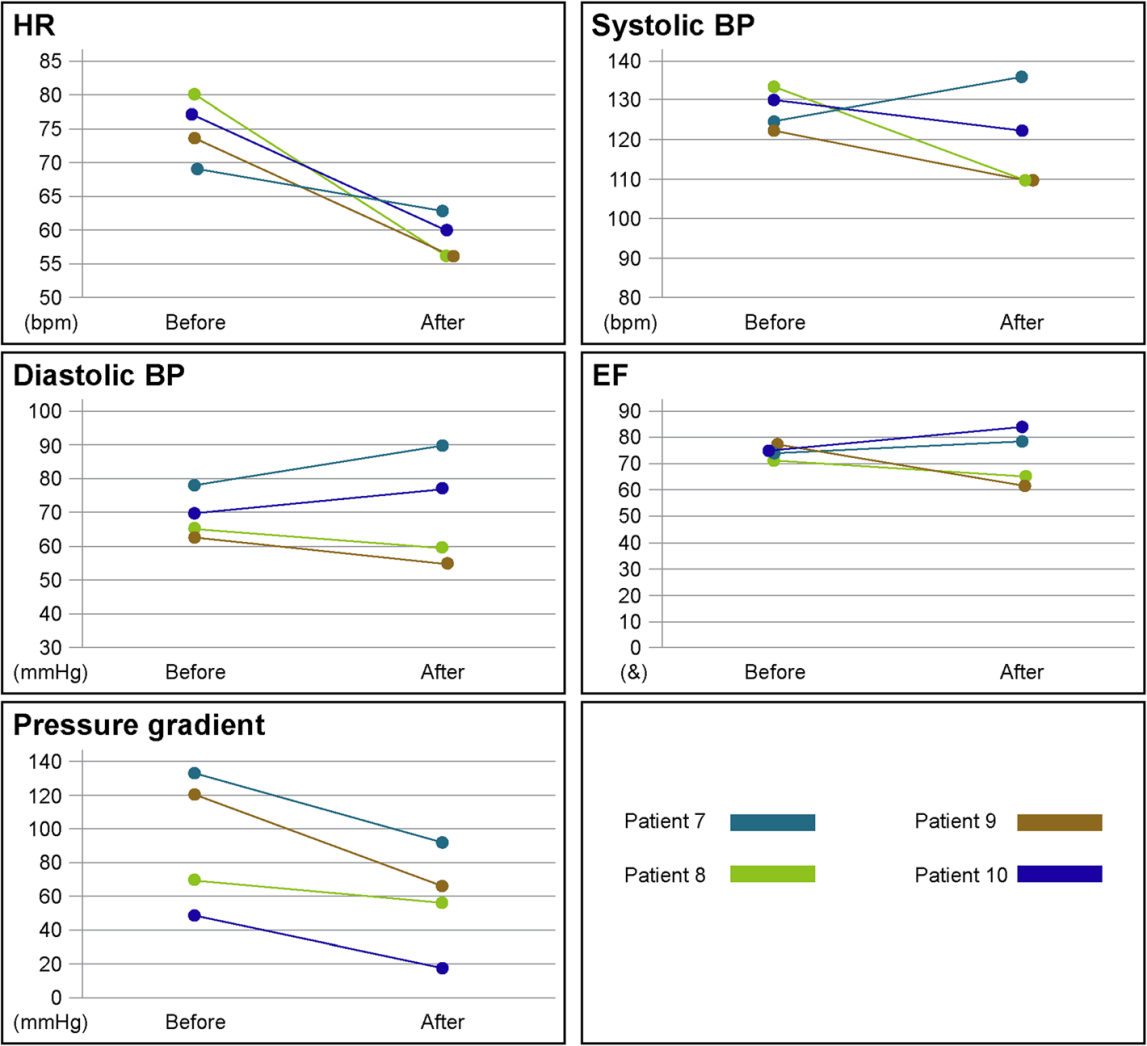
Changes of hemodynamics and echocardiographic features in patients newly introduced to the bisoprolol transdermal patch. In the add-on therapy group, 1 patient (patient 7) was newly introduced to the 8-mg bisoprolol transdermal patch and 3 patients (patient 8, patient 9, and patient 10) were newly introduced to the 4-mg bisoprolol transdermal patch. The resting pressure gradient by Doppler echocardiography is shown. HR = heart rate; BP = blood pressure; EF = ejection fraction, bpm = beats per minute

Those 4 patients tended to have decreased heart rate (from 76 [IQR: 70–79] to 58 [IQR: 56–62] bpm, *p* = 0.07) and pressure gradient (from 95 [IQR: 54–130] to 63 [IQR: 29–85] mmHg, p = 0.07) before and after the change in medication. However, there was no significant difference in the systolic blood pressure/diastolic blood pressure (from 128 [IQR: 123–132]/69 [IQR: 65–77]) to 116 [IQR: 110–133]/69 [IQR: 57–87] mmHg, *p* = 0.27/*p* = 0.58) or LVEF (from 75 [IQR: 72–77] to 72 [IQR: 62–83]%, *p* = 0.72) (Fig. 3).

### Cardiovascular complications during the perioperative period

No cardiovascular complications occurred during the perioperative period.

Although 1 case of symptomatic hypotension occurred at the time of induction of anesthesia, which immediately improved after appropriate infusion, there were no cases of uncontrolled shock and complications of heart failure and fatal arrhythmia.

## Discussion

The main findings of the present study were as follows. First, we showed novel perioperative management of HOCM using the bisoprolol transdermal patch and described the hemodynamic and echocardiographic features before and after oral bisoprolol was switched to transdermal patch therapy or transdermal patch therapy was started as a new β-blocker medication. Second, we implemented perioperative care without serious adverse events. To the best of our knowledge, this is the first report to demonstrate the utilization of the bisoprolol transdermal patch in patients with HOCM.

### Dose conversion rate from oral bisoprolol to the bisoprolol transdermal patch and precise timing of drug switching

There was no significant change in heart rate, blood pressure, LV contraction, and the LV pressure gradient before and after switching the medication. Therefore, we concluded that the basic dose conversion rate from oral bisoprolol to the bisoprolol transdermal patch was appropriate in our study. In fact, a previous clinical study that switched from oral bisoprolol to the bisoprolol transdermal patch using the same dose conversion rate herein in patients with chronic heart failure showed equivalent efficacy and good tolerability without significant safety concerns [8]. The present study included one patient who presented with severe heart failure and fluid retention. In this case, the pharmacological effect of the bisoprolol transdermal patch was stronger than that of the oral bisoprolol, and since one episode of symptomatic hypotension occurred, dose adjustment was required. Intestinal edema due to severe heart failure causes abrupt absorption from the oral medication, which can explain this patient’s condition. In such situations, the blood level concentration can be relatively decreased with oral medication than with transdermal medication. Only 1 patient was included in the study with a high baseline dose of oral bisoprolol (7.5 mg/day) in the present study. It would be interesting to study a larger population taking higher doses of oral β-blocker. Moreover, the trough value at 96 h after starting bisoprolol transdermal administration was constant and reached a steady state. Therefore, we recommend that the drug be switched at more than 96 h before non-cardiac surgery [8, 9]. We also recommend that physicians measure the pressure gradient at more than 96 h after drug switching when they assess the LVOT and mid-ventricular obstruction.

### Continuation of β-blockers and the withdrawal syndrome

The patients who underwent gastrointestinal operation or required tracheal intubation for operation were unable to continue taking oral β-blockers. The withdrawal syndrome in perioperative care of non-cardiac surgery was reported in patients who had already been prescribed a β-blocker [14]. In that situation, a rebound increase in heart rate was observed when the admiration of the β-blocker was promptly stopped, and this can induce myocardial ischemia, arrhythmia, or heart failure [15]. A previous study also reported that transient β-adrenergic hypersensitivity occurs after β-blocker withdrawal in HOCM and is associated with significant physiologic changes and adverse clinical consequences [16]. Patients with HOCM potentially can deteriorate into a critical condition when the regular medication is rapidly stopped. Therefore, guidelines recommended the continuation of β-blockers in periprocedural management for preventing cardiovascular complications [2, 3, 5, 13]. Physicians need to keep the medication regular in order to maintain a consistent blood level in patients with HOCM. Moreover, transdermal drug delivery provides another advantage over oral admiration in terms of the constant release of the drug for long periods [17, 18]. Thus, patients with HOCM are likely candidates for the transdermal drug delivery system.

### Heart rate control of atrial fibrillation during perioperative care

Since patients with HOCM also more frequently deteriorate into a critical condition with atrial fibrillation than those without HOCM, adequate control of heart rate is necessary with a β-blocker as a first-line agent in most HOCM patients with atrial fibrillation [19]. Thus far, intravenous preparation has been preferred to oral preparations for the management of atrial fibrillation during perioperative or intensive care. Internationally, an esmolol injection is frequently used [20], whereas landiolol is commonly used in intensive care in Japan [21]. However, these β-blockers are costly. In addition to these drugs, the bisoprolol transdermal patch can be an alternate treatment option. A retrospective analysis of 16 patients with critical illness complicated with rapid atrial fibrillation were switched from a landiolol injection to the bisoprolol transdermal patch; the switching of medication was conducted safely with appropriate heart rate control; additionally, the bisoprolol transdermal patch was able to lower the cost of β-blockers by over $1000 per patient [22].

### Underestimation of pressure gradient during perioperative care

Herein, the parameter of TTE was measured at rest but not under stress (exercise), and the pressure gradient by echocardiography at rest was controlled below 30 mmHg in some patients. However, even if the pressure gradient seemed to be relatively low at rest, many patients with HOCM presented with a substantial pressure gradient during exercise [23]. Hence, in the perioperative period, when hemodynamics change dynamically, the pressure gradient is inferred to be increased in the present study.

The present study had several limitations. First, because of the single-center study design, the number of cases was limited, and a comparison of transdermal patch therapy and standard oral bisoprolol therapy was not performed in our study. Second, all data analyses were performed retrospectively. Third, we might have underestimated the efficacy of β-blockers because we included some patients who were not very symptomatic (New York Heart Association classes I and IIs) or patients who experienced improvement in their severe symptoms after the previous intervention. Forth, the groups in our study showed heterogeneity. This may make it difficult to assess the drug effect as an individual entity. A prospective study comparing oral bisoprolol and bisoprolol transdermal patch should be performed.

## Conclusions

We described the utilization of the bisoprolol transdermal patch during perioperative care for non-cardiac surgery in patients with HOCM. We determined that the hemodynamic features of these patients did not change significantly after switching to patch therapy. Further, initiation of the bisoprolol transdermal patch as a new ß-blocker medication tended to decrease the pressure gradient. This unique approach could be an alternate management strategy for HOCM. However, a prospective comparison study is needed to confirm our results.

## Supplementary information

**Additional file 1.** : Detailed baseline characteristics of each patient.

## Data Availability

The datasets used and/or analysed during the current study are available from the corresponding author on reasonable request.

## Abbreviations

HOCM: Hypertrophic obstructive cardiomyopathy
HCM: Hypertrophic cardiomyopathy
IQR: Interquartile range
LVEF: Left ventricular ejection fraction
LVOT: Left ventricular outflow tract
PTSMA: Percutaneous transluminal septal myocardial ablation
TTE: Transthoracic echocardiography
MRI: Magnetic resonance imaging
LV: Left ventricular
bpm: Beats/min
EF: Ejection fraction
